# dupRadar: a Bioconductor package for the assessment of PCR artifacts in RNA-Seq data

**DOI:** 10.1186/s12859-016-1276-2

**Published:** 2016-10-21

**Authors:** Sergi Sayols, Denise Scherzinger, Holger Klein

**Affiliations:** 1 Bioinformatics Core Facility, Institute of Molecular Biology, Ackermannweg 4, 55128 Mainz, Germany; 2 Technische Hochschule Bingen, Berlinstraße 109, Bingen am Rhein, 55411 Germany; 3 Target Discovery Research, Boehringer Ingelheim Pharma GmbH & Co KG, Birkendorferstraße 67, 88397 Biberach an der Riß, Germany

**Keywords:** RNA-Seq, PCR artefacts, Duplication rate, Single cell RNA-Seq, Bioconductor, Quality control tool

## Abstract

**Background:**

PCR clonal artefacts originating from NGS library preparation can affect both genomic as well as RNA-Seq applications when protocols are pushed to their limits. In RNA-Seq however the artifactual reads are not easy to tell apart from normal read duplication due to natural over-sequencing of highly expressed genes. Especially when working with little input material or single cells assessing the fraction of duplicate reads is an important quality control step for NGS data sets. Up to now there are only tools to calculate the global duplication rates that do not take into account the effect of gene expression levels which leaves them of limited use for RNA-Seq data.

**Results:**

Here we present the tool dupRadar, which provides an easy means to distinguish the fraction of reads originating in natural duplication due to high expression from the fraction induced by artefacts. dupRadar assesses the fraction of duplicate reads per gene dependent on the expression level. Apart from the Bioconductor package dupRadar we provide shell scripts for easy integration into processing pipelines.

**Conclusions:**

The Bioconductor package dupRadar offers straight-forward methods to assess RNA-Seq datasets for quality issues with PCR duplicates. It is aimed towards simple integration into standard analysis pipelines as a default QC metric that is especially useful for low-input and single cell RNA-Seq data sets.

**Electronic supplementary material:**

The online version of this article (doi:10.1186/s12859-016-1276-2) contains supplementary material, which is available to authorized users.

## Background

### Sources of duplicate reads in Next-Generation sequencing

Next Generation Sequencing has become a standard assay for many questions in molecular biology. It involves the preparation of sequencing libraries out of fragments of DNA or RNA molecules and sequencing adapters, PCR amplification and sequencing. The calculation of the fraction of duplicate reads has become a standard step for quality control in NGS experiments, as high duplication rates can hint towards problems in different steps of the NGS library preparation process. In particular, the variety of molecules that can be seen after sequencing correlates with minute amounts of input material (“molecular bottleneck”) or too many PCR cycles. This can lead to low library complexity. Furthermore overloading of a sequencing flow cell may result in optical duplicates or problems with reagents can lead to elevated duplication rates. Duplicate reads can also be caused by a combination of complex genomic loci and insufficient read length or even issues with the reference genome.

In RNA-Seq however it is common to have high overall fractions of duplicate reads not due to technical artifacts. This is known and discussed in the community (e.g. [1, 3, 4]) but is still sometimes misunderstood [2]. Often the top 5 % of expressed genes take up more than 50 % of all reads in a common RNA-Seq dataset [5]. Read counts for highly expressed genes easily surpass the threshold of 1 read per bp of the exon model, at which read duplication is inevitable. Due to a number of biases in the process of RNA-Seq [6] read duplication in RNA-Seq starts even below the 1 read per bp threshold. In RNA-Seq duplication originating from technical artifacts such as described before are confounded with natural read duplication due to highly expressed genes, hence overall duplication rate is not a suitable measure for quality control purposes.

### Effects and treatment of PCR duplicates in RNA-Seq data

In assays involving genomic DNA (e.g. resequencing, ChIP-Seq) reads marked as duplicates with tools such as the established picard [7], or the more recent bamUtil dedup [8] and biobambam [9] are commonly removed before further analyzing the data. In RNA-Seq studies with the aim to quantify expression however the situation is more complex. Duplicate reads also arise naturally in highly expressed genes, hence complete removal of duplicate reads affects estimation of expression levels. Tools such as eXpress [10] attempt to tackle related problems by smoothing the read coverage. However this approach is not applicable to situations in which systematic over-estimation of read counts on a large fraction of genes exists.

### Detection of duplicate reads in Next-Generation sequencing

Currently there are many tools available that address the overall duplication rates or read frequencies of NGS data sets [7, 11–16]. Commonly, the non-systematic detection of PCR artefacts in RNA-Seq analysis relies on the visual inspection in a genome browser, where problematic data sets show typical stacked reads in loci with low and medium expression.

Here we present dupRadar, a tool to systematically detect anomalous duplication rate profiles and simplify the task of identification of data sets that require further in-depth assessment.

## Implementation

dupRadar relates the duplication rate and length normalized read counts of every gene to model the dependency of this two variables. It requires a BAM file with mapped and duplicate marked reads, and a gene model in GTF format. Internally dupRadar calls the featureCounts function from the RSubread package [17] several times, to count all and the duplicate marked reads per genes, both uniquely as well as multi-mapping reads. Furthermore dupRadar calculates the per gene duplication rate and reads per kilobase (RPK) as a proxy for relative gene expression. The resulting calculations are stored in a data frame which can be directly passed on to different visualization functions, which show the dependence of the duplication rate on gene expression. Besides fitting a logistic model to the dependency between duplication rate and RPK, dupRadar estimates the baseline duplication rate for lowly expressed genes which can be used as an indicator for general problems inside a data set.

Additionally, the data frame can be used for further processing of the data in standard read count based differential gene expression tools [18–20], or for other purposes such as the detection of genes that are exclusively covered by multi-mapping reads.

To enable interpretation of the dependency of duplication rate and gene expression, dupRadar currently includes various visualization functions. Beyond that the vignette of the Bioconductor package contains examples for customised plots using dupRadar. For the sake of usability, it includes wrappers for some common tools for duplicate marking in order to streamline the processing of the data sets.

To demonstrate the effect of PCR artefacts also on downstream analysis we perform a simulation study based on the Airway dataset commonly used in Bioconductor courses [21] (results in Additional file 1: Figure S1). To obtain a comparable dataset with a high fraction of duplicate reads, we subsampled the reads of the original library to different fractions (50 and 10 %), and applied an amplification step to the remaining ones to match again the number of reads in the original library, thus creating simulated libraries with respectively 50 and 90 % of duplicate reads, following a Poisson process to simulate what happens in a PCR..Subsequently we perform differential expression analysis using edgeR [22] for both the original data as well as the datasets with 50 and 90 % of artificially added duplicate reads.

## Results and discussion

Recently, RNA-Seq protocols were improved considerably, leading to less technical duplicates and the linked issues. Still in our experience possible problems are worth to be checked for by default, especially if protocols are pushed to or beyond their boundaries or more recent low-input or single cell RNA-Seq protocols are used.

To demonstrate the usage of dupRadar we apply a typical work flow for selected single read RNA-Seq data sets from the study of Marinov et al. [23] ranging from single cells to cell pools to bulk RNA data. We map reads using STAR [24] and mark duplicate reads using BamUtil dedup [8]. Together with the human reference gene annotation GTF included in the iGenomes collection for the UCSC hg19 build [25], we use the resulting bam files as input for dupRadar’s duplication rate calculation function. As an example Table 1 contains the entries from a sample of 10 genes out of the full set for the library 13276 (SRR764800). We supply instructions to regenerate the results in the supplement (Additional file 2: Methods 1, Additional file 3: Methods 2, Additional file 4: Table S1.).

**Table 1:** Example values for a sample of 10 genes from the library 13276

ID	geneLength	allCounts	filteredCounts	dupRate	dupsPerId	RPK	RPKM
LOC100288069	1371	17	15	0.12	2	12.40	0.60
LINC00115	1317	28	28	0.00	0	21.26	1.03
LOC643837	9233	281	246	0.12	35	30.43	1.47
FAM41C	1706	1	1	0.00	0	0.59	0.03
LOC100130417	496	0	0	NA	0	0.00	0.00
SAMD11	2554	0	0	NA	0	0.00	0.00
NOC2L	2800	329	273	0.17	56	117.50	5.67
KLHL17	2564	2	2	0.00	0	0.78	0.04
ISG15	666	590	271	0.54	319	885.89	42.78
AGRN	7326	3	3	0.00	0	0.41	0.02

Some columns were omitted due to space constraints; refer to Additional file 7: Table S2 for the complete table

**Fig. 1:**
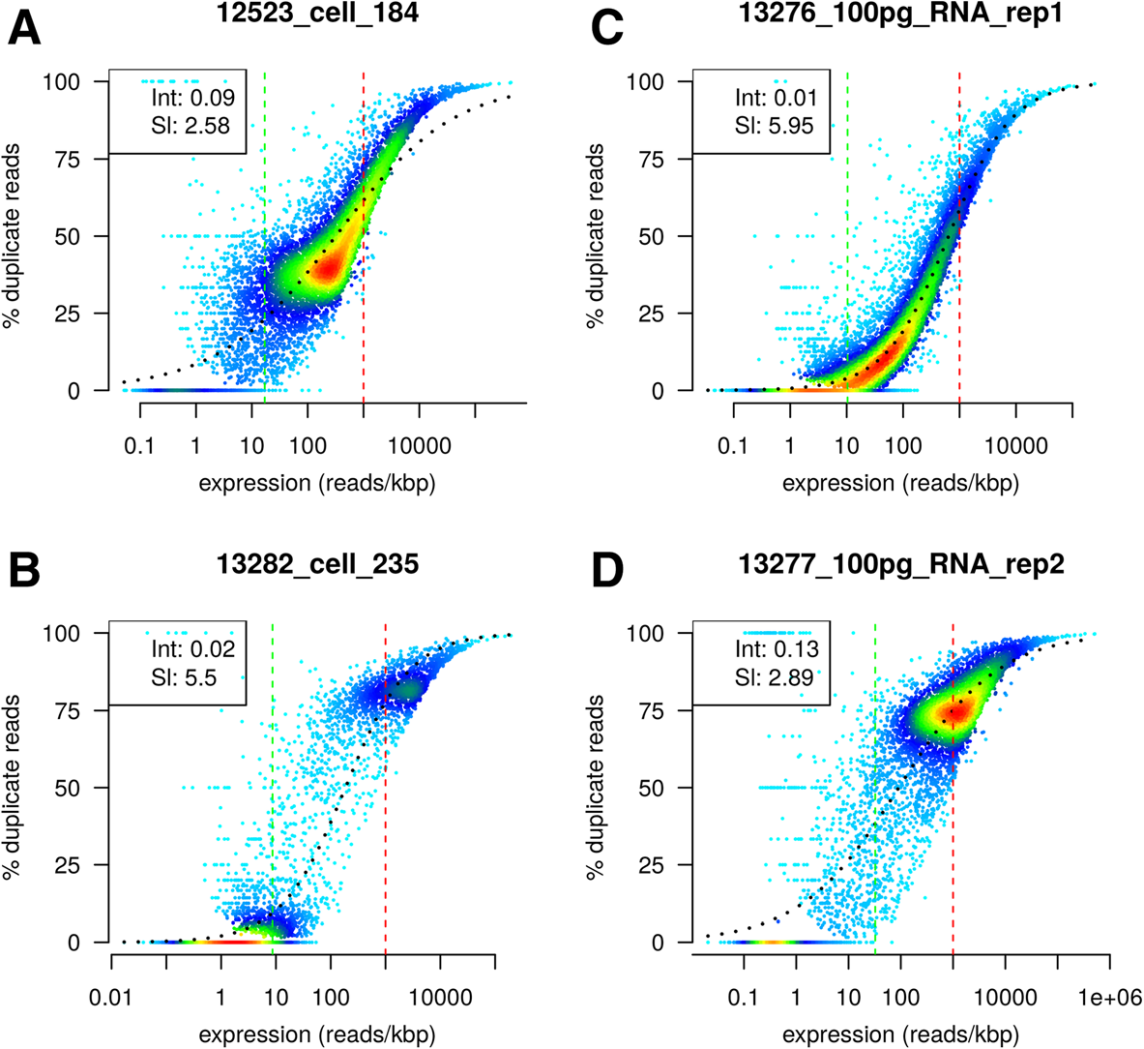
Several RNA-seq datasets from Marinov et al. [26]. Legends shows the intercept and slope of a fitted logit model. **a** Single cell experiment with relatively low duplication rates and most of the genes detected. **b** Single cell experiment with most of the genes undetected and high duplication rate on the detected ones. **c** RNA-seq experiment pushing the protocol to only 100 pg of input material, with low duplication rates and relatively good identification of genes. **d** same RNA-seq experiment, showing over-sequencing due to higher sequencing depth of the library

Based on the duplication rates, we generate the main visualizations of dupRadar in Fig. 1. The effects of over-sequencing libraries of limited complexity in cases of little input material as well as an example for a bulk RNA-Seq dataset without any traces of PCR duplicates. The given plots indicate the duplication rate in relation to the gene expression. Ideally single read RNA-Seq experiments at common read depths are expected to show low duplication rates for lowly expressed genes in the bottom left of the plot, with the duplication rate rising as the expression level approaches the 1 read/bp boundary. Beyond this threshold genes are covered almost completely with reads marked as duplicates due to their high expression levels (e.g. Fig. 1c). Data sets based on lower amount of input material show the effects of limited complexity of the library, resulting in higher duplication rates already at lowly expressed genes, leading to the majority of data points being shifted upwards to higher duplication rates also for lowly expressed genes (e.g. Fig. 1d). Similar situations can be observed for data sets with actual PCR artifacts. DupRadar does not define fixed thresholds for acceptable data quality on purpose, as PCR duplication rate can be influenced by various parameters. However already low levels of PCR artefacts can have an influence on downstream analysis and interpretation of data.

Although paired-end libraries facilitate the distinction between duplicates due to adding the fragment length as an extra variable to distinguish molecules, the problem is not completely solved. For typical dupRadar plots of paired-end libraries see Additional file 5: Figure S2. The recent introduction of unique molecular identifiers (UMI) during library preparation, allows for exact distinction of technical and biological duplicates and therefore also the removal of technical duplicates [24], which alleviates the described problem on the side of experimental procedures.

To assess the impact of excess PCR amplification on downstream analysis in RNA-Seq studies we simulated data sets with defined amounts of PCR artifacts (Additional file 1: Figure S1 and Additional file 6: Methods 3) based on good quality original data [21], and subsequently performed differential expression both on the original data as well as the data with simulated PCR problems. While there is a large overlap of 1199 genes that are differentially expressed in both the good and the bad data, the analysis shows that PCR artefacts introduce both high numbers of false positive (124) and false negative (720) differentially expressed genes.

Choice of the aligner as well as of the reference annotation both influence read mapping, quantification and downstream analyses in RNA-Seq experiments [27, 28]. On gene level, differences between aligner and annotation can also be observed in dupRadar results, however globally in our experience the assessment of library quality does not differ depending on the these parameters. We recommend not to make the choice of read mapper or reference annotation dependent on the dupRadar step.

## Conclusions

The Bioconductor package dupRadar offers straight-forward methods to assess RNA-Seq datasets for problems with duplicate reads and is aimed towards simple integration into standard analysis pipelines as a default QC metric.

While dupRadar serves as a diagnostics method for PCR duplicates, we regard the issue of correction for these artefacts as yet unsolved, with a potential to extend dupRadar with correction functions. Currently we advise colleagues to treat with caution RNA-seq data strongly affected by technical duplicates and repeat library preparation and sequencing if possible. Furthermore the simulation results suggest that even consistent levels of PCR artifacts over all samples of a project do not cancel out and may lead to wrong conclusions in the downstream analysis of data.

Similar effects comparable to over-sequencing of highly expressed genes are implicated for certain types of enrichment-based assays (e.g. ChIP-Seq of a specific transcription factor with high read-depths). Suitability of dupRadar in this area remains to be explored.

## Availability and requirements

**Project name**: dupRadar

**Project home page**: http://bioconductor.org/packages/dupRadar/

**Operating system(s)**: Linux; MacOS

**Programming language**: R > = 3.2

**Other requirements**: Bioconductor > = 3.2

**License**: GNU GPL

**Any restrictions to use by non-academics**: None

## Additional files

Additional file 1: Figure S1. Simulation results with 50 % of duplicates. (PDF 3904 kb)
Additional file 2: Methods 1. Additional description of analysis of single cell data, differences between SR and PE libraries, and effect of PCR bottleneck on differential expression. (DOCX 18 kb)
Additional file 3: Methods 2. Instructions to reproduce main images. (MD 4 kb)
Additional file 4: Table S1. Mapping statistics of the data set used to generate Fig. 1. (CSV 520 bytes)
Additional file 5: Figure S2. Simulation results with 90 % of duplicates. (PDF 3130 kb)
Additional file 6: Methods 3. Simulation of datasets and differential expression. (RMD 7 kb)
Additional file 7: Table S2. Full version of Table 1 from manuscript. (CSV 2183 kb)

## Abbreviations

bp: Base pair
ChIP: Chromatin immunoprecipitation
NGS: Next-generation sequencing
PCR: Polymerase chain reaction
QC: Quality control
RPK: Reads per kilobase
UMI: Unique molecular identifiers
