# Supplementary figures and images for: dupRadar: a Bioconductor package for the assessment of PCR artifacts in RNA-Seq data
Source: BMC Bioinformatics. 2016 Oct 21;17:428. doi: 10.1186/s12859-016-1276-2 (PMC5073875; doi:10.1186/s12859-016-1276-2)

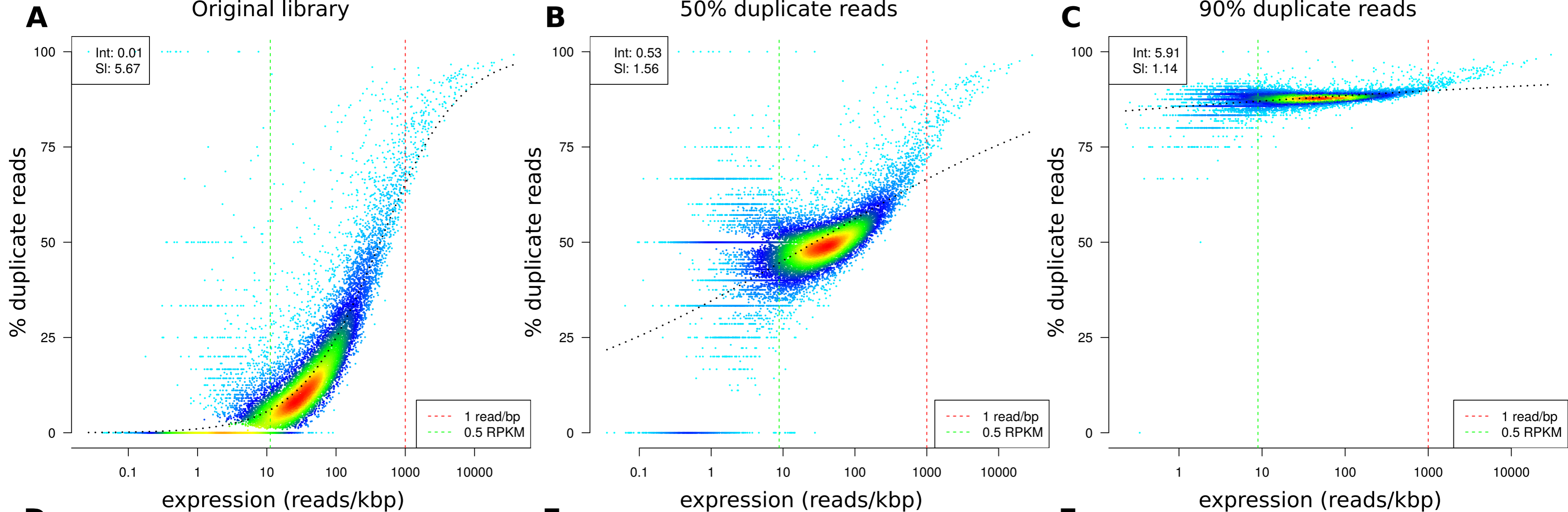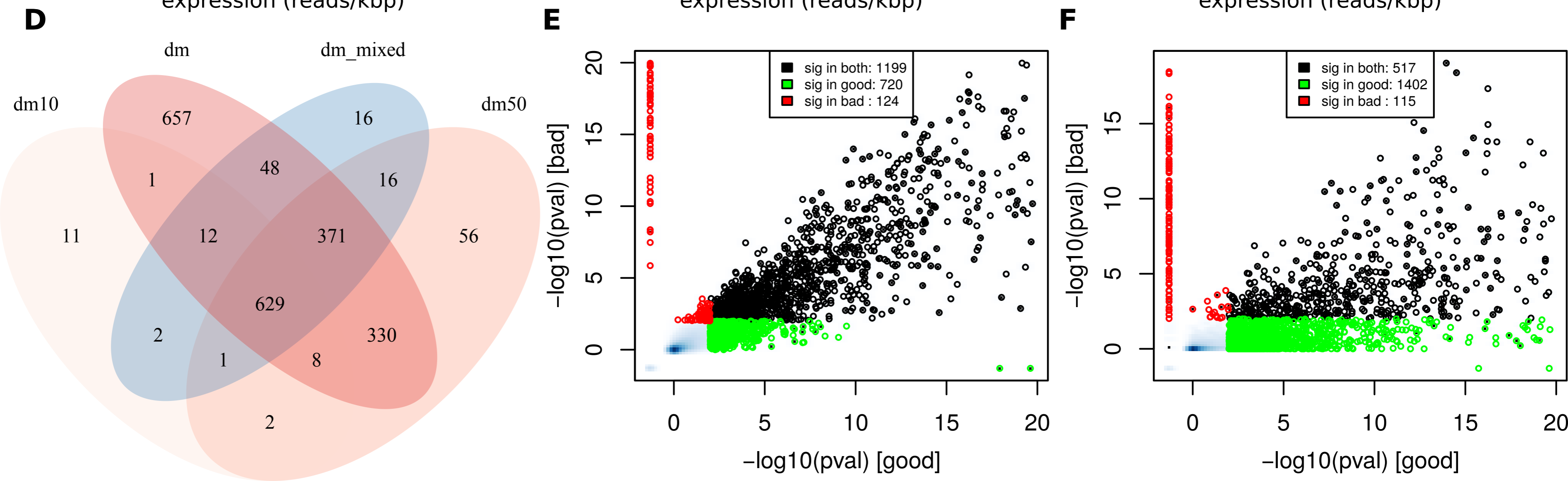

Supplement: Additional file 1: Figure S1. — Simulation results with 50 % of duplicates. (PDF 3904 kb) [file 12859_2016_1276_MOESM1_ESM.pdf]

**A**

Paired-end library

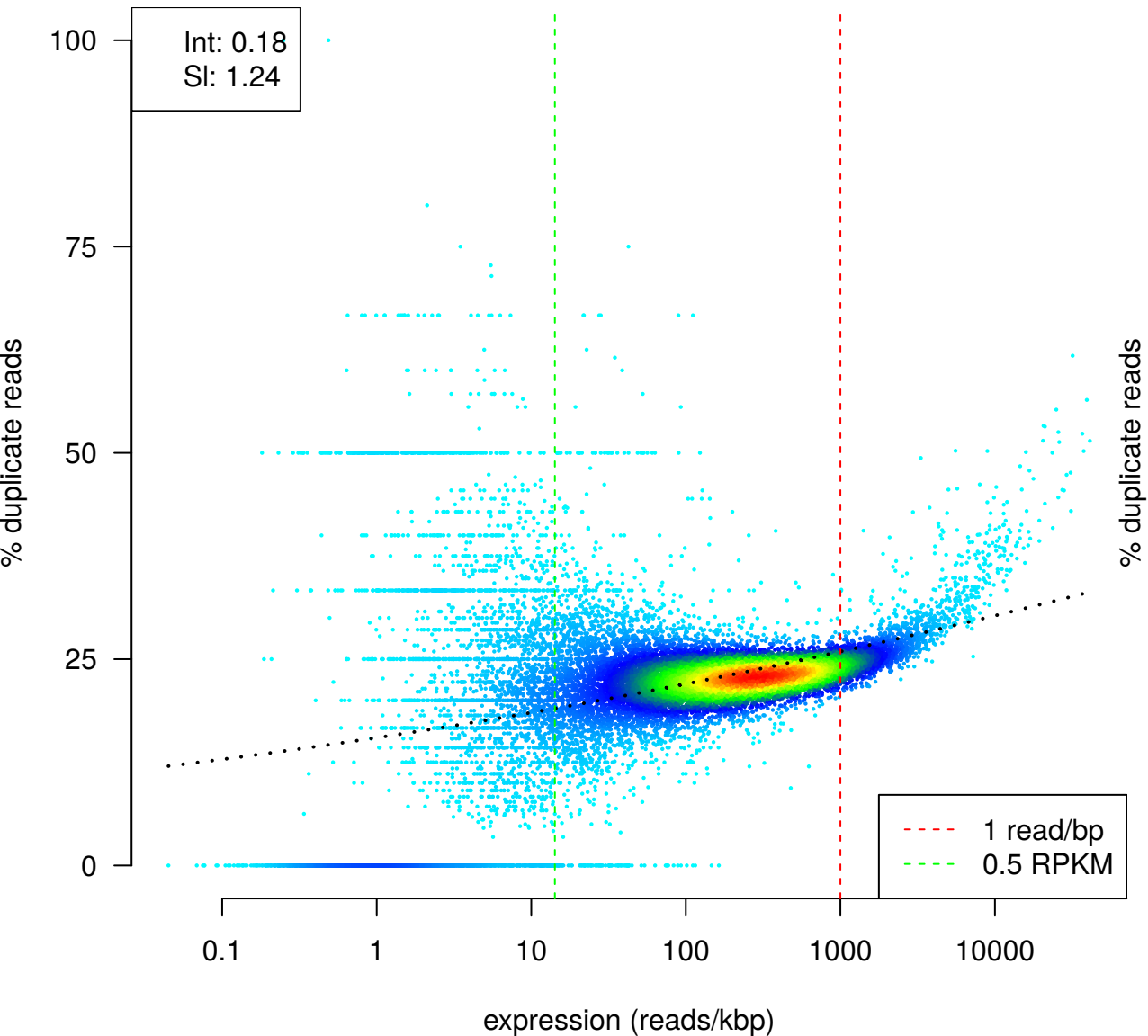**B**

Only first read of the paired-end library

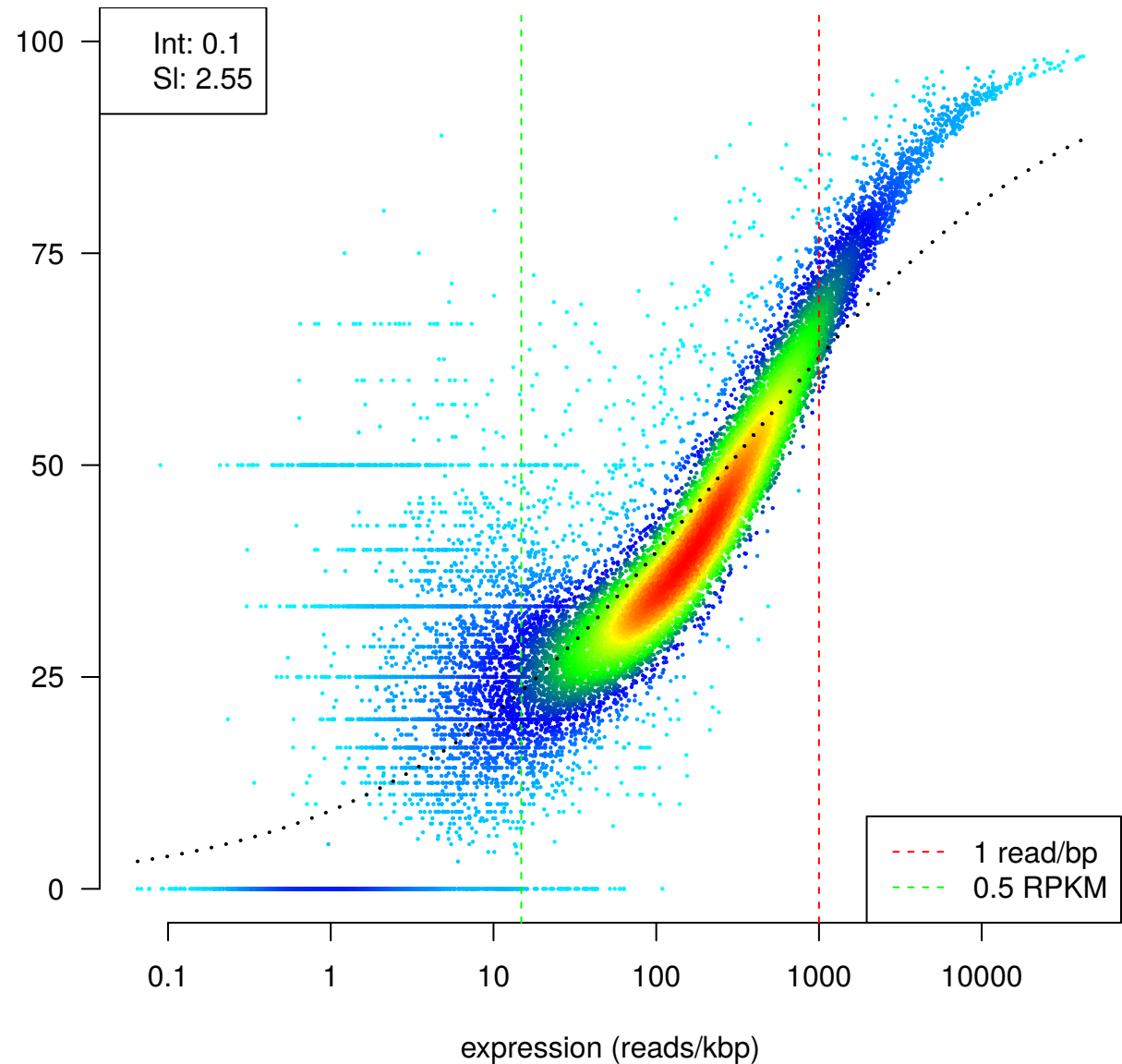

Supplement: Additional file 5: Figure S2. — Simulation results with 90 % of duplicates. (PDF 3130 kb) [file 12859_2016_1276_MOESM5_ESM.pdf]
