# Supplementary material for: dupRadar: a Bioconductor package for the assessment of PCR artifacts in RNA-Seq data
Source: BMC Bioinformatics. 2016 Oct 21;17:428. doi: 10.1186/s12859-016-1276-2 (PMC5073875; doi:10.1186/s12859-016-1276-2)
Supplement: Additional file 2: — Methods 1. Additional description of analysis of single cell data, differences between SR and PE libraries, and effect of PCR bottleneck on differential expression. (DOCX 18 kb) [file 12859_2016_1276_MOESM2_ESM.docx]

Supplementary methods

Single cell data from Marinov et al. (2014)

We attach a markdown document with the step by step instructions to recreate the results discussed in the article, together with a brief description of them.

To process the data from Marinov et al. (2014), first we retrieved the raw data from GEO (GSE44618) using the SRA toolkit[1]. The resulting fastq files were aligned using STAR version 2.4.0h[2] and duplicates marked with the bamUtil[3] dedup function.

The duplicate information was processed using dupRadar, calculating the matrices with counts and creating the plots as indicated in the supplementary instructions (additional markdown document).

Differential expression analysis on libraries with different PCR bottleneck

To perform the simulation study based on the Airway dataset, we downloaded the rawdata from GEO (GSE52778) and proceeded with subsampling with samtools [6] to only 50% and 10% of the original reads. Then, we followed with an amplification step in order to simulate different amounts of input material, based on a Poisson process to simulate what happens in a PCR. The final simulated libraries contained a similar amount of reads with the original libraries, thus containing 50% and 90% of duplicates respectively, simulating different degrees of affection.

To perform differential expression analysis, we used edgeR [7] for both the original data as well as the datasets with 50% and 90% of artificially added duplicate reads. We also called differential expression analysis on a mix of libraries with different levels of affection, constructing the 2 groups by randomly sampling from the original libraries and the simulated ones.

Refer to the differential expression details in the additional Rmd file.

***Supp. Fig. 1*** *Effects on the identification of differentially expressed genes in the same library with different simulated ratios of duplicated reads. Supp fig 1A dupradar plot of duplication rates of the original library, naturally affected by duplicates. Supp fig 1B dupradar plot of the same library with simulated 50% of duplicated reads. Supp fig 1C dupradar plot of the same library with simulated 90% of duplicated reads. Supp fig 1D Venn diagram with the agreement of the differentially expressed (DE) genes, pairwise comparing a group of replicates with the 3 simulated conditions, plus a forth one coming from a mix of these 3, with a reference condition. Supp fig 1E comparison of the pvalues of the DE analysis between the original libraries and the libraries simulating 50% duplicated reads. Supp fig
19 comparison of the pvalues of the DE analysis between the original libraries and the libraries simulating 90% duplicated reads.*

UHRR-HBRR datasets to compare paired end and single read libraries

We downloaded from the Illumina’s BaseSpace (<https://basespace.illumina.com/datacentral>) public data repository, the TopHat aligned data from the “HiSeq 4000: RNA-Seq 64-plex (MAQC HBRR and UHRR)” project. This project contains the alignment, assembly and differential expression analysis using the Tuxedo suite[4] of 32 samples distributed in 2 groups, human brain (HBRR) and universal human reference (UHRR) from the MAQC consortium.

From these samples, we marked duplicate reads with bamUtils dedup and performed duplication analysis with dupRadar as described before. Then, we extracted the first read only and repeated the analysis. The final aim was to compare paired end and single read libraries.

***Supp. Fig. 2*** *Due to additional information of the second read paired-end libraries show less natural duplication even for highly expressed genes than single-read libraries. A) dupRadar plot on PE library. B) dupRadar plot on the same dataset, only taking into account the first read.*

Supplementary references

[1] Wheeler DL, Barrett T, Benson DA, Bryant SH, Canese K, Chetvernin V, Church DM, Dicuccio M, Edgar R, Federhen S, Feolo M, Geer LY, Helmberg W, Kapustin Y, Khovayko O, Landsman D, Lipman DJ, Madden TL, Maglott DR, Miller V, Ostell J, Pruitt KD, Schuler GD, Shumway M, Sequeira E, Sherry ST, Sirotkin K, Souvorov A, Starchenko G, Tatusov RL, Tatusova TA, Wagner L, Yaschenko E.: Database resources of the National Center for Biotechnology Information. Nucleic Acids Res. 2008 Jan; 36 (Database issue): D13-21. Epub 2007 Nov 27. PMID: 18045790

[2] Dobin A, Davis CA, Schlesinger F, Drenkow J, Zaleski C, Jha S, Batut P, Chaisson M, Gingeras TR. STAR: ultrafast universal RNA-seq aligner. Bioinformatics. 2013 Jan 1;29(1):15-21. doi: 10.1093/bioinformatics/bts635. Epub 2012 Oct 25.

[3] BamUtil Dedup, a repository that contains several programs that perform operations on SAM/BAM files, <http://genome.sph.umich.edu/wiki/BamUtil>, Accessed 19. Oct. 2015

[4] Trapnell C, Roberts A, Goff L, Pertea G, Kim D, Kelley DR, Pimentel H, Salzberg SL, Rinn JL, Pachter L. Differential gene and transcript expression analysis of RNA-seq experiments with TopHat and Cufflinks. Nat Protoc. 2012 Mar 1;7(3):562-78. doi: 10.1038/nprot.2012.016.

[5] Himes BE, Jiang X, Wagner P, Hu R, Wang Q, Klanderman B, Whitaker RM, Duan Q, Lasky-Su J, Nikolos C, Jester W, Johnson M, Panettieri R Jr, Tantisira KG, Weiss ST, Lu Q. “RNA-Seq Transcriptome Profiling Identifies CRISPLD2 as a Glucocorticoid Responsive Gene that Modulates Cytokine Function in Airway Smooth Muscle Cells.” PLoS One. 2014 Jun 13;9(6):e99625. PMID: 24926665. GEO:GSE52778.

[6] Li H, Handsaker B, Wysoker A., Fennell T., Ruan J., Homer N., Marth G., Abecasis G., Durbin R. and 1000 Genome Project Data Processing Subgroup (2009) The Sequence alignment/map (SAM) format and SAMtools. Bioinformatics, 25, 2078-9. [PMID: 19505943]

[7] Robinson MD, McCarthy DJ and Smyth GK (2010). edgeR: a Bioconductor package for differential expression analysis of digital gene expression data. Bioinformatics 26, 139-140.
